# An Improvement in the Antimicrobial Resistance Patterns of Urinary Isolates in the Out-Of-Hospital Setting following Decreased Community Use of Antibiotics during the COVID-19 Pandemic

**DOI:** 10.3390/antibiotics12010126

**Published:** 2023-01-09

**Authors:** Sara Tedeschi, Elena Sora, Andrea Berlingeri, Denis Savini, Elena Rosselli Del Turco, Pierluigi Viale, Fabio Tumietto

**Affiliations:** 1 Department of Medical and Surgical Sciences, University of Bologna, 40138 Bologna, Italy; 2 Infectious Diseases Unit, Department for Integrated Infectious Risk Management, IRCCS Azienda Ospedaliero Universitaria di Bologna, 40138 Bologna, Italy; 3 Pharmacy Department, Bologna Local Health Unit, 40138 Bologna, Italy; 4 Antimicrobial Stewardship Unit, Bologna Local Health Unit, 40138 Bologna, Italy; 5 Microbiology Unit, IRCCS Azienda Ospedaliero Universitaria di Bologna, 40138 Bologna, Italy

**Keywords:** antibiotic consumption, primary care, COVID-19

## Abstract

After the onset of COVID-19 pandemic, a decrease in antibiotic consumption in the out-of-hospital setting was observed. However, data about the impact of this reduction on antimicrobial resistance are lacking. The aim of this study was to assess antibiotic consumption and antibiotic resistance at the community level in an Italian province before and after the beginning of the COVID-19 pandemic. We carried out an observational study, comparing antibiotic consumption in the community during 2019 and 2020 and the antibiotic resistance patterns of *Enterobacterales* cultured from urine samples from the out-of-hospital setting during the first semester of 2020 and 2021. Overall, antibiotic consumption decreased by 28% from 2019 to 2020 (from 13.9 to 9.97 DDD/1000 inhabitants/day). The main reductions involved penicillins (ATC J01C, from 6.9 to 4.8 DDD/1000 inhabitants/day, −31%), particularly amoxicillin/clavulanate (ATC J01CR02, −30%) and amoxicillin (J01CA04, −35.2%). Overall, 6445 strains of *Enterobacterales* were analyzed; in 2020, the susceptibility rate of amoxicillin/clavulanate increased from 57.5% to 87% among isolates from the primary care setting (*p* < 0.001) and from 39% to 72% (*p* < 0.001) among isolates from LTCF. The reduction in the community use of antibiotics observed in 2020 was followed by a change in the antimicrobial resistance patterns of urinary isolates.

## 1. Introduction

The COVID-19 pandemic occurred during global efforts to combat the spread of antimicrobial resistance [1,2] and caused an unprecedented disruption of healthcare systems, with a possible negative impact on antibiotic consumption and antimicrobial resistance. Indeed, concerns about secondary bacterial infections in patients hospitalized with COVID-19 increased the clinical workload for healthcare personnel, the subsequent interruption of antimicrobial stewardship activities, and recommendations for the use of azithromycin during the earlier stage of the pandemic. The shift to virtual healthcare could have increased the use of antibiotics, both in the hospital setting and in primary care [3,4]. Indeed, many studies have reported an increase in hospital antibiotic consumption after the onset of the COVID-19 pandemic [5]. On the contrary, in primary care, several developed countries reported a decrease in antibiotic consumption, especially during the first wave of the pandemic [3,6]. However, data are scarce compared with those available for the hospital setting, and information about the impact of this reduction on antimicrobial resistance is lacking. 

The aim of this study was to assess antibiotic consumption and antibiotic resistance at the community level in an Italian province before and after the beginning of the COVID-19 pandemic. 

## 2. Results

### 2.1. Antibiotic Consumption

We assessed community antibiotic consumption in an Italian province during 2019 (the pre-pandemic period) and during 2020 (the pandemic period). The consumption of antibacterials for systemic use (ATC J01) observed during 2020 was 9.97 DDD/1000 inhabitants/day, with a decrease of 28% compared with consumption in 2019 (13.9 DDD/1000 inhabitants/day). 

The main reductions, expressed in DDD/1000/inhabitants/day, involved penicillins (ATC J01C, from 6.98 to 4.8, −31%), particularly amoxicillin/clavulanate (ATC J01CR02, from 5.3 to 3.7, −30%) and amoxicillin (J01CA04, from 1.64 to 1.06, −35.2%), and other beta-lactams (J01D, from 1.48 to 0.97, −34%) (Table 1). 

With respect to fluoroquinolones (J01MA), the reduction started in 2019 and continued in 2020 (1.73, 1.15 and 0.81 DDD/1000 inhabitants/day in 2018, 2019 and 2020, respectively). This finding is a consequence of: (i) a specific antimicrobial stewardship intervention directed toward general practitioners in the Bologna province carried out during 2018 and 2019, (ii) the recommendations of the national plan to contrast antimicrobial resistance to reduce the out-of-hospital use of fluoroquinolones by 10% from 2016 to 2020 [7], and (iii) the safety warnings issued by the FDA recommending limiting the use of fluoroquinolones [8]. 

The reduction the use of macrolides was driven mainly by the reduction in the use of clarithromycin. Indeed, during the first phase of the pandemic (i.e., March and April 2020) characterized by great therapeutic uncertainty, azithromycin was used in the treatment of patients diagnosed with COVID-19 in combination with hydroxychloroquine [9]. Therefore, during this period, the consumption of this antibiotic increased, both in the community and in the hospital setting and, even though this therapeutic approach was abandoned following the results of a randomized trial [10], the reduction in the use of this antibiotic has been contained.

We also observed a reduction in the use of all other antibiotic classes, except for tetracycline, including those prescribed less frequently (i.e., aminoglycosides and trimethoprim-sulfamethoxazole).

### 2.2. Microbiology 

Overall, 6445 strains of *Enterobacterales* were analyzed for the purposes of this study: 5887 from the primary care setting and 558 from long-term care facilities (LTCF). In particular, during the first semester of 2020, 2544 and 427 strains were identified from the primary care setting and LTCFs, respectively, while during the first semester of 2021, 3343 and 131 strains were identified from primary care and LTCF, respectively.

**Figure 1:**
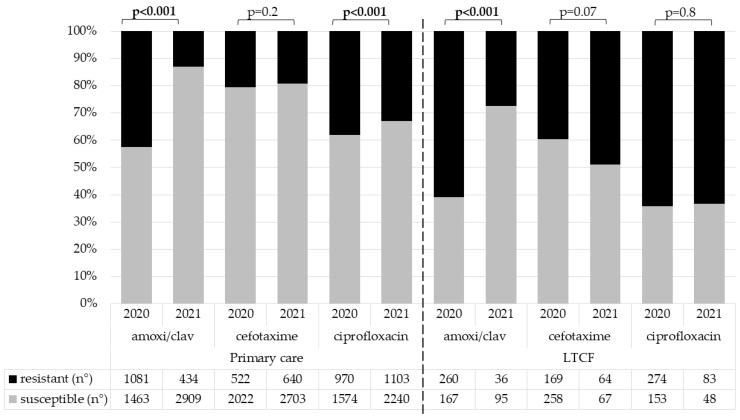
Comparison of antimicrobial susceptibility of *Enterobacterales* cultured from urine samples collected in the out-of-hospital setting during the first semester of 2020 and 2021.

The susceptibility to penicillins improved significantly among strains isolated during 2021, with the rate of susceptibility to amoxicillin/clavulanate increasing from 57.5% to 87% among isolates from the primary care setting (*p* < 0.001) and from 39% to 72% (*p* < 0.001) among isolates from LTCFs (Figure 1). We also observed an improvement in the susceptibility to fluoroquinolones only among isolates from primary care, while resistance to third-generation cephalosporines remained stable and was about 20% in primary care and more than 40% in LTCFs.

## 3. Discussion

In this study, we have described the changes in the antimicrobial resistance patterns of urinary isolates coming from the out-of-hospital setting, following the reduction in the community use of antibiotics observed during the first phase of the COVID-19 pandemic in an Italian province. To our knowledge, this is the first report of the impact of a reduction in the consumption of antibiotics in the community on antibiotic resistance. 

The first phase of the COVID-19 pandemic had a dramatic impact on healthcare systems, involving both the hospital and the community level. In hospitals, the overall antibiotic consumption increased following the onset of the pandemic, with different trends over time. In the first months, there was an increase in the consumption of amoxicillin-clavulanate, ceftriaxone and azithromycin, corresponding to a phase with a high hospital admission rate from the community and empirical antibiotic coverage of all cases of COVID-19-related pneumonia. Subsequently, many patients with severe disease and colonization or infections with MDROs accumulated in the hospital, especially in the ICU, and the consumption of broad-spectrum antibiotics (including carbapenems, daptomycin, linezolid and novel cephalosporin-β-lactamase inhibitor combinations) increased [11,12].

On the contrary, despite reasonable concerns, the dreaded increase in antibiotic consumption during the COVID-19 pandemic did not occur in primary care. Instead, the containment measures applied to control COVID-19 (lockdown, social distancing and the widespread use of masks) probably reduced the circulation of other respiratory viruses (e.g., influenza), which could lead to the prescription of antibiotics. In addition, the decrease in ambulatory visits for mild illnesses may have had a role in the reduction in antibiotic use in the community. Indeed, several studies describing a reduction in the use of antibiotics in the community during the first wave of COVID-19 pandemic in high-income countries have been published.

In the United States, there was an estimated 33% decline in the dispensing of antimicrobials between January and May 2020, with the greatest impact in April and May 2020 [13]. The reduction affected all groups of antimicrobials, except for azithromycin, which had a 5% increase from February to March 2020, with a subsequent decrease in the next months [13]. 

In Australia, a reduction in the dispensing of antimicrobials was also observed from March to May 2020, mainly affecting antibiotics used in the treatment of respiratory tract infections. Subsequently, the dispensing of antimicrobials increased, although it did not reach pre-pandemic values [14]. 

A Dutch study comparing the follow-up data of prescriptions for outpatients described a decrease in pneumonia, mastoiditis, pyelonephritis and gastrointestinal infections, as well as in antimicrobial treatments [15].

In Andalusia, Spain, a study comparing antibiotic use in the community during the first and second quarters of 2019 and 2020 showed a 7.6% decrease in prescribing antimicrobials. This decrease occurred for all antimicrobials except azithromycin, which remained stable over the study period [16].

In addition, an Italian study described the impact of the COVID-19 lockdown on antibiotic consumption in the Emilia-Romagna Region, comparing consumption in March, April and May 2020 with the same months of the previous two years, focusing on the use of azithromycin use. The rates of consuming antibacterials for systemic use (ATC J01) in the general population showed a deviation of −22%, −44% and −53% in March, April and May 2020, respectively, compared with the average rates registered in the same months in 2018–2019. The rates of azithromycin consumption (ATC J01FA10) in March, April and May 2020 had a deviation of +23%, −2% and −48%, respectively, compared with the averages registered in the same months in 2018–2019 [6].

Our data, which are representative of the local level, are consistent with those reported in the literature. Indeed, we also observed a reduction in the overall antibiotic consumption in the community, with the partial exception of azithromycin. Indeed, during the very first phases of the pandemic, a widely accepted therapeutic approach to COVID-19 included the use of azithromycin in combination with hydroxychloroquine; however, this approach was based on insufficient evidence and was then discouraged. 

Our data on antibiotic consumption refer to a longer period than the abovementioned studies, as we included antibiotic consumption for all of 2020, confirming a sustained reduction in community antibiotic consumption, without the rebound observed in Australia. This favorable trend has been confirmed at the national level by the Italian report on antibiotic use [17].

It is expected that this change in prescribing antimicrobials following the onset of COVID-19 will have an impact on antimicrobial resistance [18,19]. However, the results of the reduction in antibiotic use observed in primary care were not obvious. It is well known that a high increase in antimicrobial consumption leads to a rapid increase in resistance, although a decrease in consumption does not translate into a rapid decrease in resistance, as described in Kronoberg County, Sweden, where a 24-month voluntary restriction in the use of trimethoprim-containing drugs was not followed by a reduction in the resistance of *E. coli* to this drug [20,21].

Moreover, the lower selection density of the out-of-hospital setting and the interconnection of the healthcare system could have compromised the possible favorable impact on antibiotic resistance. Indeed, to our knowledge, no studies have been published so far showing that the decrease in antimicrobial consumption during the COVID-19 pandemic in primary care has had a positive effect on resistance in community pathogens.

Our study showed an improvement in the antimicrobial resistance patterns of common pathogens causing urinary tract infections following the reduction in antibiotic consumption in the out-of-hospital setting during the COVID-19 pandemic. This is an important finding, because our results highlight that it is possible to reverse the unfavorable trend toward increased antimicrobial resistance through prudent antibiotic use, contrary to what was described in the abovementioned Swedish experience [20,21]. In our study, we described an important reduction in the overall volume of antibiotic use, mainly driven by a reduction in the use of amoxicillin/clavulanate, a drug which is prone to inappropriate prescription in primary care (e.g., for upper respiratory tract infections), rapidly followed by lower resistance to this antibiotic. A possible explanation for this finding may be found in the high level of baseline resistance (approximately 40% in primary care and 60% in LTCF), probably driven by the unnecessary use of this antibiotic in the community. As a consequence, these results could be used to emphasize the importance of implementing antimicrobial stewardship activities to reduce unnecessary antibiotic use in primary care.

Implementing actions to improve the prescription of antibiotics in primary care is very important to combat the spread of antimicrobial resistance, as most antibiotic prescriptions are related to care received outside the hospital. However, some barriers exist to the effective implementation of antimicrobial stewardship programs in the out-of-hospital setting, such as a lack of specific expertise and dedicated personnel, pressure from pharmaceutical marketing, and patients and caregivers’ expectations about prescribing antimicrobials, which may be stronger than during hospital care [22]. Furthermore, especially in primary care, even with a well-structured and effective antimicrobial stewardship intervention, it would be very difficult to see a rapid and measurable impact on strong indicators of the outcome (e.g., patients’ clinical outcomes, antibiotic resistance) to motivate healthcare practitioners to continue their antimicrobial stewardship efforts. 

Our study has several limitations. First, it involved one Italian province, and the results may have been influenced by the local epidemiology and antimicrobial prescription patterns, limiting the possibility of generalizing the results. Second, we assessed only the antimicrobial susceptibility patterns of urinary isolates, excluding other important community pathogens such as respiratory pathogens (e.g., *Streptococcus pneumoniae*, *Haemophilus influenzae*), which could have suffered the consequences of the modified prescription patterns. The level of macrolide resistance in *S. pneumoniae* could have increased following the widespread use of azithromycin [3]; however, this phenomenon has been limited over time and the overall use of macrolides has finally reduced. Third, the reduction in antibiotic use could be transient, and its effects may not be sustained; however, to date, antibiotic consumption in the out-of-hospital setting in Italy has remained lower than in the pre-pandemic period, and prospective surveillance of antimicrobial resistance is warranted.

To conclude, COVID-19 has presented a unique opportunity for reducing antibiotic use in primary care, which allowed us to assess that reduced antibiotic use has had a positive impact on the antimicrobial resistance patterns of pathogens circulating in the community. These results should be used to reinforce the importance of appropriate prescribing of antibiotics in primary care, and our efforts should be directed to maintaining these achievements.

## 4. Materials and Methods

We carried out an observational, retrospective study in an Italian province with approximately 1 million inhabitants. We compared antibiotic consumption in the community during 2019 and 2020, and the antibiotic resistance patterns of Enterobacteriaceae cultured from urine samples sent to the microbiology department from the out-of-hospital setting during the first semester (from 1 January to 30 June) of 2020 and 2021, respectively. 

The data sources were the regional drug database of outpatient prescriptions reimbursed by the Regional Health Service and the local microbiology database. 

Antibiotics were classified according to the Anatomical Therapeutic Chemical (ATC) classification system of the World Health Organization [20]. Antibiotic consumption was calculated as the defined daily dose (DDD) per 1000 inhabitants/day for antibacterials for systemic use (ATC J01) and for antibiotic classes categorized as penicillins with an extended spectrum (J01CA), combinations of penicillins including beta-lactamase inhibitors (ATC J01CR), macrolides (ATC J01FA), fluoroquinolones (ATC J01MA), cephalosporins (ATC J01DB; J01DC; J01DD; J01DE) and others (all other ATC J01). 

Urine cultures were processed according to the national guidelines of the AMCLI (Italian Clinical Microbiologists’ Association). Identification was performed using MALDI-TOF (matrix-assisted laser desorption ionization—time of flight, Bruker Corporation) and susceptibility testing was performed using a Vitek-2 automated system (bioMérieux). The results were interpreted in accordance with the clinical breakpoints of the European Committee on Antimicrobial Susceptibility Testing [23].

The differences in the rates of antimicrobial resistance were compared using the chi-square test. Differences were considered statistically significant in case of a *p* value < 0.05. 

## Figures and Tables

**Table 1:** Antibiotic use in the out-of-hospital setting during the study period (DDD/1000 inhabitants/day).

Antibiotic Class	2020	2019	2018	∆2019–2020
J01—Antibacterials for systemic use	9.97	13.9	14.1	−28%
J01C—Penicillins	4.8	6.98	6.75	−31%
J01CR02—Amoxicillin/clavulanate	3.7	5.3	5.1	−30%
J01CR04—Amoxicillin	1.06	1.64	1.62	−35%
J01D—Other beta-lactams	0.97	1.48	1.43	−34%
J01DD—Third generation cephalosporines	0.92	1.4	1.35	−34%
J01MA—Fluoroquinolones	0.8	1.15	1.73	−30%
J01FA—Macrolides	2.09	2.99	2.98	−30%
J01FA09—Clarithromycin	1.02	1.77	1.81	−42%
J1FA10—Azithromycin	1.03	1.11	1.09	−10%
J01AA—Tetracyclines	0.35	0.36	0.32	0.3%
J01GB—Aminoglycosides	0.009	0.011	0.014	−14%
J01EE01—Trimethoprim-sulfamethoxazole	0.44	0.48	0.41	−9%

## Data Availability

Not applicable.
